# Genomics and cellulolytic, hemicellulolytic, and amylolytic potential of *Iocasia fonsfrigidae* strain SP3-1 for polysaccharide degradation

**DOI:** 10.7717/peerj.14211

**Published:** 2022-10-19

**Authors:** Sobroney Heng, Sawannee Sutheeworapong, Verawat Champreda, Ayaka Uke, Akihiko Kosugi, Patthra Pason, Rattiya Waeonukul, Ruben Michael Ceballos, Khanok Ratanakhanokchai, Chakrit Tachaapaikoon

**Affiliations:** 1 School of Bioresources and Technology, King Mongkut’s Institute of Technology Thonburi, Bangkok, Thailand; 2 Pilot Plant Development and Training Institute, King Mongkut’s Institute of Technology Thonburi, Bangkok, Thailand; 3 National Center for Genetic Engineering and Biotechnology, Thailand Science Park, Klong Luang, Pathumthani, Thailand; 4 Biological Resources and Post-harvest Division, Japan International Research Center for Agricultural Sciences, Ibaraki, Japan; 5 Excellent Center of Enzyme Technology and Microbial Utilization, Pilot Plant Development and Training Institute, King Mongkut’s Institute of Technology Thonburi, Bangkok, Thailand; 6 Department of Biological Sciences, University of Arkansas, Fayetteville, AR, United States of America; 7 Arkansas Center for Space & Planetary Sciences, University of Arkansas, Fayetteville, AR, United States of America

**Keywords:** Halophilic alkaliphilic anaerobic bacterium, Cellulolytic enzyme, Hemicellulolytic enzyme, Amylolytic enzyme, Carbohydrate-binding module, *Iocasia fonsfrigidae*

## Abstract

**Background:**

Cellulolytic, hemicellulolytic, and amylolytic (CHA) enzyme-producing halophiles are understudied. The recently defined taxon *Iocasia fonsfrigidae* consists of one well-described anaerobic bacterial strain: NS-1^T^. Prior to characterization of strain NS-1^T^, an isolate designated *Halocella* sp. SP3-1 was isolated and its genome was published. Based on physiological and genetic comparisons, it was suggested that *Halocella* sp. SP3-1 may be another isolate of *I. fronsfrigidae*. Despite being geographic variants of the same species, data indicate that strain SP3-1 exhibits genetic, genomic, and physiological characteristics that distinguish it from strain NS-1^T^. In this study, we examine the halophilic and alkaliphilic nature of strain SP3-1 and the genetic substrates underlying phenotypic differences between strains SP3-1 and NS-1^T^ with focus on sugar metabolism and CHA enzyme expression.

**Methods:**

Standard methods in anaerobic cell culture were used to grow strains SP3-1 as well as other comparator species. Morphological characterization was done via electron microscopy and Schaeffer-Fulton staining. Data for sequence comparisons (*e.g.*, 16S rRNA) were retrieved via BLAST and EzBioCloud. Alignments and phylogenetic trees were generated via CLUTAL_X and neighbor joining functions in MEGA (version 11). Genomes were assembled/annotated via the Prokka annotation pipeline. Clusters of Orthologous Groups (COGs) were defined by eegNOG 4.5. DNA-DNA hybridization calculations were performed by the ANI Calculator web service.

**Results:**

Cells of strain SP3-1 are rods. SP3-1 cells grow at NaCl concentrations of 5-30% (w/v). Optimal growth occurs at 37 °C, pH 8.0, and 20% NaCl (w/v). Although phylogenetic analysis based on 16S rRNA gene indicates that strain SP3-1 belongs to the genus *Iocasia* with 99.58% average nucleotide sequence identity to *Iocasia fonsfrigida* NS-1^T^, strain SP3-1 is uniquely an extreme haloalkaliphile. Moreover, strain SP3-1 ferments D-glucose to acetate, butyrate, carbon dioxide, hydrogen, ethanol, and butanol and will grow on L-arabinose, D-fructose, D-galactose, D-glucose, D-mannose, D-raffinose, D-xylose, cellobiose, lactose, maltose, sucrose, starch, xylan and phosphoric acid swollen cellulose (PASC). D-rhamnose, alginate, and lignin do not serve as suitable culture substrates for strain SP3-1. Thus, the carbon utilization profile of strain SP3-1 differs from that of *I. fronsfrigidae* strain NS-1^T^. Differences between these two strains are also noted in their lipid composition. Genomic data reveal key differences between the genetic profiles of strain SP3-1 and NS-1^T^ that likely account for differences in morphology, sugar metabolism, and CHA-enzyme potential. Important to this study, *I. fonsfrigidae* SP3-1 produces and extracellularly secretes CHA enzymes at different levels and composition than type strain NS-1^T^. The high salt tolerance and pH range of SP3-1 makes it an ideal candidate for salt and pH tolerant enzyme discovery.

## Introduction

Starch-based biomass, such as brewery-spent grains, cassava pulp, rice bran, sago pith residues, and wheat bran, is a by-product of agro-industrial and agricultural operations (Hoang & Nghiem, 2021). Starch-based biomass is produced in significant amounts around the world. For example, more than 174.1 MT/year of sugarcane, cassava, rice, and palm are produced in Thailand alone (Jusakulvijit, Bezama & Thrän, 2021). These feedstocks are composed of polysaccharides, including: cellulose, hemicellulose, and starch, which serve as low-price raw materials for bioproducts. Such starch-based biomass is hydrolyzed by cellulolytic, hemicellulolytic, and amylolytic (CHA) enzymes to yield monosaccharides and oligosaccharides (Cheawchanlertfa et al., 2021). Monosaccharides can be converted to bioethanol, organic acids, or other value-added products while oligosaccharides can be used as prebiotics (Phakeenuya et al., 2020).

Anaerobic bacteria are a proven natural source for the identification and isolation of novel CHA enzymes (Cheawchanlertfa et al., 2021). The bioprospecting of extremophiles, including halophilic anaerobic bacteria, has also yielded novel enzymes with unique properties for commercial applications (Kivistö & Karp, 2011). In this study, we examine CHA enzymes derived from the halophilic anaerobic bacterium designated as *Halocella* sp. SP3-1 (Heng et al., 2019), which is renamed as described below.

*Iocasia* was recently proposed as a new genus with *I. fonsfrigidae* NS-1^T^ as the archetype (Zhang et al., 2021). This strain was isolated from cold seep sediment of the South China Sea. *I. fonsfrigidae* NS-1^T^ was shown to metabolize several carbohydrates, including: starch, xylan, alginate, carboxymethyl cellulose, and a polymer of the aromatic compound lignin (Zhang et al., 2021). *I. fonsfrigidae* NS-1^T^ is a moderate halophile, which will readily grow in 1.25–15.0% NaCl.

Prior to reporting the discovery of *I. fonsfrigidae* NS-1^T^, our laboratory published the complete genome of an isolate originally designated as *Halocella* sp. SP3-1 (Heng et al., 2019), which we identify and rename in the present study as *Iocasia fonsfrigidae* strain SP3-1. The strain SP3-1 was isolated from the soil of a salt evaporation pond (13°28′37.55″N; 100°7′8.27″E) in the Samut Sakhon province of Thailand (Heng et al., 2019). SP3-1 readily grows on cellulose, hemicellulose, or starch under higher salt content (5–30% NaCl) than strain NS-1^T^. The complete genome sequences for *I. fonsfrigidae* strains SP3-1 and NS-1^T^ were analyzed. The average nucleotide identity (ANI) between the two strains is 97.64% (Zhang et al., 2021), supporting the “same species” determination based on a suggested ANI threshold of 95–96% (Richter & Rosselló-Móra, 2009). Although, we conclude that the SP3-1 isolate is a strain of *I. fonsfrigidae*, genetic, physiological, and biochemical properties between SP3-1 and NS-1^T^ vary. For example, NS-1^T^ encodes several genes not found in SP3-1, including genes encoding for proteins related to carbohydrate metabolism, ABC transporters, PTS sugar transporters, type II secretion systems, type I-B and type III-B CRISPR associated proteins, and clusters of proteins related to ethanolamine and propanediol metabolism (Zhang et al., 2021). Conversely, strain SP3-1 exhibits higher salt tolerance and encodes genes not found in strain NS-1^T^. These include genes that code for endo- *β*-1,4-galactanase, xylan- *α*-1,2-glucuronosidase, *β*-xylosidase, *α*-L-arabinofuranosidase, and *β*-L-arabinofuranosidase (*i.e.,* hemicellulolytic enzymes) and oligo- *α*-1,6-glucosidase (amylolytic enzymes). The unique CHA enzymes constituency of the strain SP3-1 proteome motivate this study. Specifically, we distinguish strains SP3-1 and NS-1^T^ based on phylogenetics, physiology, and biochemistry with particular interest in differentiating the respective proteomes based on enzymes content. Our analysis demonstrates that *I. fonsfrigidae* strain SP3-1 expresses a suite of CHA enzymes with potential for optimal functionality under high salinity conditions.

## Material and Methods

### Bacterial strains and media

Strain SP3-1 was isolated from the soil sample (Heng et al., 2019), whereas *Halocella cellulosilytica* DSM 7362^T^ was purchased from the Leibniz Institute, DSMZ-German Collection of Microorganisms and Cell Cultures GmbH. Strain SP3-1 was deposited at Thailand Institute of Scientific and Technological Research Culture Collection (TISTR) and Korean Collection for Type Cultures (KCTC) under accession numbers TISTR 2992 and KCTC 25333, respectively. Strain SP3-1 was cultured in the basal medium, pH 8.0 (BM) composed of (per liter): 200 g NaCl, 1.5 g KH_2_PO_4_, 2.9 g K_2_HPO_4_, 2.1 g urea, 4.5 g yeast extract, 0.5 g cysteine-HCl, 0.001 g resazurin, and 200 µL mineral solution (25.0 g/L MgCl_2_^**.**^6H_2_O, 37.5 g/L CaCl_2_^**.**^2H_2_O and 0.3 g/L FeSO_4_^**.**^6H_2_O). For shorter term storage, strain SP3-1 was preserved at −20 °C in liquid media with 25% of glycerol. Two methods were used for longer term storage: storages at −80 °C in liquid media with 25% of glycerol and *via* lyophilization.

Strain *H. cellulosilytica* DSM7362^T^ was cultured in DSMZ medium 702 (pH 7.0) (https://www.dsmz.de/microorganisms/medium/pdf/DSMZ_Medium702.pdf). Both media were anaerobically prepared in bottles sealed with butyl rubber stoppers under an atmosphere of high-purity N_2_ and sterilized by autoclaving at 121 °C for 15 min. Phosphoric acid swollen cellulose (PASC), prepared from Avicel PH-101, as previously described by Zhang et al. (2006), xylan, and starch were used as the sole carbon source to observe the ability of bacteria to degrade cellulose, hemicellulose (*i.e.,* xylan), and starch, respectively.

### 16S rRNA gene analysis and phylogenetic tree

Genomic DNA was extracted as previously described by Heng et al. (2019). The 16S rRNA gene was amplified *via* PCR with the universal primers 8F (5′-AGAGTTTGATCCTGGCTCAG-3′) and 1492R (5′-GGTTACCTTGTTACGACTT-3′) (Chimtong et al., 2014). The PCR amplification was performed with 1 µL of a DNA template, followed by 5 µL of 10X Ex-Taq buffer, 1 µL of 10 mM dNTPs, 1 µL of each 10 µM primer, and 0.25 µL of Ex-Taq DNA polymerase (Promega Corp., Madison, WI, USA). PCR conditions consisted of an initial denaturation step at 95 °C for 30 s, followed by 30 cycles at 95 °C for 30 s, annealing at 68 °C for 30 s, and extension at 68 °C for 1 min. The final extension step was 10 min at 68 °C. PCR product was purified by a QIAquick PCR purification kit (QIAGEN, Hilden, Germany). The nearly complete 16S rRNA gene sequence was compiled with the BioEdit software (Hall, Biosciences & Carlsbad, 2011). The 16S rRNA gene sequence of strain SP3-1 was compared to taxa which were retrieved through BLAST (Altschul et al., 1997) and EzBioCloud databases (Yoon et al., 2017). The 16S rRNA sequence of strain SP3-1 and correlated taxa were aligned by *via* CLUTAL_X in MEGA software version 11 and phylogenetic trees were constructed using the neighbor-joining (NJ) method (Tamura, Stecher & Kumar, 2021). Confidence values for phylogenetic tree branches were determined *via* bootstrap analyses on 1,000 replicates (Rzhetsky & Nei, 1992).

### Physiological and biochemical analysis

Cells of strain SP3-1 were harvested during late exponential growth phase (3 days). Cell cultures were filtered through a nucleopore membrane filter (0.6 µm pore size), dehydrated by a series of graded ethanol solutions, and dried to the critical point with liquid CO_2_ (Pason, Kyu & Ratanakhanokchai, 2006). Samples were then examined under a scanning electron microscope (SEM, SU800 Hitachi, Japan) to identify morphological features, such as flagella (Belas & Colwell, 1982). Bacterial motility was assessed by colony growth characteristics on semi-solid media (0.4% agar plates) *via* microscopy. Gram staining was performed as previously described (Manafi & Kneifel, 1990) and spore formation was examined using the Schaeffer-Fulton staining method (Oktari et al., 2017). Physiological characterization of strain SP3-1 was performed using standard protocols commonly used in bacterial systems (Zhang et al., 2021; Doetsch, 1981). For all experiments, cells were incubated at various temperatures under static conditions in triplicate. The growth range and optima of strain SP3-1 were monitored *via* absorbance spectroscopy (*e.g.*, optical density readings) at 600 nm in anaerobic Hungate tubes under various conditions, including a range of: temperatures 25–70 °C (25, 37, 40, 45, 50, 55, 60, 65, 70 °C); pH of 5.0–10.0 (pH 5.0, 6.0, 7.0, 8.0, 9.0, 10.0); and, NaCl concentrations of 0–30% (w/v) (0, 2.5, 5, 7.5, 10, 15, 20 and 30%).

System pH was controlled by varying buffer systems. For pH 5.0–6.5, an acetate buffer was used. A MOPS buffer was used for pH 6.5–8.0. A TABS buffer was used for pH 8.0–9.0. A CHES buffer was used for pH 9.0–10.0. Optical density (OD_600nm_) readings were taken every 24 hrs for three days until achieving the late exponential phase. Fermentation products were identified *via* a gas chromatograph equipped with a flame ionization detector and a Carbopack B-DA/4% Carbowax 20M column (GC-14A; Shimadzu, Japan). The column, injector, and detector temperatures were 170 °C, 230 °C, and 230 °C, respectively. Carbon source use was tested by growing strain SP3-1 in BM containing 0.5% (w/v) of one the following: L-arabinose, D-fructose, D-galactose, D-glucose, D-mannose, D-raffinose, D-rhamnose, D-xylose, cellobiose, lactose, maltose, sucrose, PASC, xylan, starch, alginate, or lignin.

### Chemotaxonomic analysis

The cell wall peptidoglycan was determined as described by Komagata & Suzuki (1987). Cellular fatty acids were extracted, methylated, and analyzed using the standard microbial identification system protocol (Sherlock Microbial Identification System, version 6.1), whereas fatty acids were identified using the TSBA6 database of the microbial identification system (Sasser, 1990). Polar lipids were analyzed from freeze-dried cells by two-dimensional thin-layer chromatography (TLC), as described by Minnikin et al. (1984). Appropriate detection reagents were used for visualizing TLC bands: phosphomolybdic acid reagent 5% (w/v) solution in ethanol (Sigma-Aldrich, Saint Louis, MO, USA) was used to detect total polar lipids; ninhydrin reagent (0.2% solution; Sigma-Aldrich Saint Louis, MO, USA) was used to detect amino lipids; the Dittmer and Lester reagent (molybdenum blue, 1.3%; Sigma-Aldrich Saint Louis, MO, USA) was used to detect phospholipids; and, Dragendorff’s reagent (Sigma-Aldrich Saint Louis, MO, USA) was used to detect phosphatidylcholine.

### Cultivation and enzyme production

Strain SP3-1 was cultivated in 1 L of BM containing 0.5% (w/v) PASC for 3 days at 37 °C, pH 8.0 under static conditions in an anaerobic chamber (Bactron II, USA). The culture supernatant was collected by centrifugation at 10,000×*g* for 15 min at 4 °C. Culture supernatant was subsequently concentrated using a hollow fiber cartridge with a 10 kDa cutoff membrane (GE Healthcare, USA). The retentate (approximately 40-times concentrated) was then used as the crude enzyme.

### Enzyme assays and protein determination of crude enzyme of *I. fonsfrigidae* strain SP3-1

Enzyme activity was determined using 50 µL of enzyme (containing 250 µg protein) mixed with 50 µL of substrate in a 50 mM sodium phosphate buffer (pH 7.0) incubated at 50 °C for 15 min. Enzymatic activity on 1% (w/v) PASC, birchwood xylan, or soluble starch were assayed by determining the amount of reducing sugar by the DNS method (Hu et al., 2008). One unit (U) of enzyme activity is defined as the amount of enzyme releasing 1 µmol of reducing sugar in 1 min. The Lowry method was used for measurement of the protein concentration and using bovine serum albumin as a standard (Lowry et al., 1951).

### Library preparation and genome sequencing of *I. fonsfrigidae* strain SP3-1

Genome sequencing and library preparation were performed as described in Heng et al. (2019). Briefly, strain SP3-1 was cultured at 37 °C in BM containing 1% (w/v) cellobiose and 20% (w/v) NaCl until late exponential growth phase under anaerobic conditions. Cell culture was collected and used for genomic DNA extraction *via* the DNeasy blood and tissue kit (Qiagen, Hilden, Germany). Subsequently, the SMRTbell template prep kit 1.0 (Pacific Biosciences, Menlo Park, CA, USA) was used to construct sequencing libraries. Polymerase reads were trimmed using high-quality regions, with a minimum subread length (500 bp), a minimum polymerase read quality (0.80), and a minimum polymerase read length (100 bp).

### Genome assembly and annotation of *I. fonsfrigidae* strain SP3-1

Genome annotation was conducted with a Prokka annotation pipeline (Seemann, 2014). The rRNA and tRNA genes were identified with the RNAmmer (Lagesen et al., 2007) and the Aragorn software (Laslett & Canback, 2004). Functional classification of protein-coding genes in strain SP3-1 was done by assigning Cluster of Orthologous Groups (COGs) codes to each gene, using eggnog-Mapper (Huerta-Cepas et al., 2017) and eggNOG version 4.5 (Huerta-Cepas et al., 2016). The *in silico* DNA-DNA hybridization (DDH) calculation between *I. fonsfrigidae* strain SP3-1 and *I. fonsfrigidae* NS-1^T^ was done using the Genome-to-Genome Distance Calculator (GGDC 3.0) (Rigden & Fernández, 2022). Online tools predict genome-to-genome distances between pairs of completely or partially sequenced genomes. Average Nucleotide Identity (ANI) was performed by the ANI Calculator web service (https://www.ezbiocloud.net/tools/ani) (Yoon et al., 2017).

### Comparative genome analysis with comparison of CHA enzyme and carbohydrate-binding module (CBM) genes between *I. fonsfrigidae* strain SP3-1 and *I. fonsfrigidae* NS-1^**T**^

The *I. fonsfrigidae* SP3-1 genome (NCBI GenBank accession number CP032760) was compared with closely related strain *I. fonsfrigidae* NS-1^T^ (NCBI GenBank accession number CP046640). The polysaccharide-degrading enzyme-related genes of strain SP3-1 were grouped into CHA enzyme-encoding and CBM-encoding families using HMMER hmmsearch with Pfam_Is HMMs (full-length models) to identify complete matches to the family, which were named per the CAZy nomenclature scheme (Cantarel et al., 2009). All hits with E-values below 10^−4^ were calculated and their sequences were further analyzed. For CHA enzymes and CBM families, which currently do not have the Pfam HMM, representative sequences were selected from the CAZy website per Warnecke et al. (2007). In this case, BLAST (http://www.ncbi.nlm.nih.gov/BLAST/) was used to identify these CHA enzymes, CBM families, and percent nucleotide sequence identity of all genes.

## Results

### 16S rRNA analysis of *Halocella* sp. SP3-1 suggests relatedness to *I. fonsfrigidae* NS-1^**T**^

**Figure 1:**
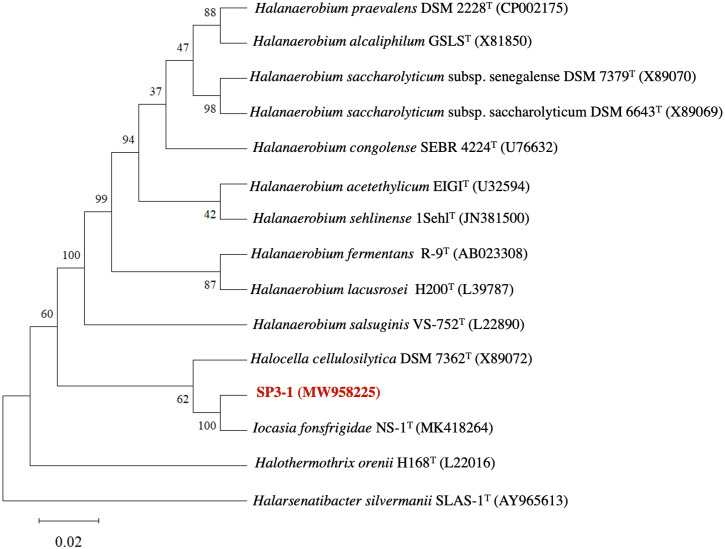
Phylogenetic tree of *I. fonsfrigidae* strain SP3-1, with other members in the genera *Halanaerobium, Halocella, Halothermothrix, Halarsenatibacter,* and *Iocasia*. The phylogenetic tree is based on comparing a nearly complete 16S rRNA g.

The genetic profile of *I. fonsfrigidae* isolate SP3-1 was examined by 16S rRNA sequence analysis. Phylogenetic analysis of 16S rRNA indicates SP3-1 falls within genus *Iocasia* of the family Halanaerobiaceae, order Halanaerobiales. *I. fonsfrigidae* NS-1^T^ (Zhang et al., 2021), *H. cellulosilytica* DSM 7362^T^ (Simankova et al., 1993), and *Halothermothrix orenii* H168^T^ (Cayol et al., 1994) are all closely related to strain SP3-1 as indicated by 99.58%, 92.77%, and 90.61% nucleotide sequence identity, respectively (Fig. 1).

Analysis of 16S rRNA genes from isolates SP3-1, NS-1^T^, and other species in the family Halanaerobiaceae indicate that isolate SP3-1 may be the same species as strain NS-1^T^ based on a 98.65% threshold for species determination (Kim et al., 2014). This agrees with a recent report noting isolates SP3-1 and NS-1^T^ as geographic variants of the same species (Zhang et al., 2021). Although 16S rRNA is used to determine relationships between genera, 16S analysis alone is not sufficient for making a same species call in microbiology (Lee et al., 1998).

### Comparative genome analysis of *I. fonsfrigidae* NS-1^**T**^ and isolate SP3-1 suggests they are geographic variants of the same species

Whole genome sequences were used to examine relatedness between isolates SP3-1 and NS-1^T^. Whole genomic sequence analysis shows 97.64% of ANI, which corresponds to a previously reported value (Zhang et al., 2021). DNA-DNA hybridization (DDH) was also conducted to further explore the relatedness between isolate SP3-1 and NS-1^T^. The *in-silico* DDH values between NS-1^T^ and isolate SP3-1 (79.9%) exceeded the accepted threshold of 70% for a distinct species call. Based on sequence similarity (Kim et al., 2014) and DDH (Qin et al., 2014), our results also indicate that NS-1^T^ and SP3-1 are the same species. Further data support these results. Specifically, *I. fonsfrigidae* NS-1^T^ was reported to have a 3,926,493 base pair (bp) genome and a total G+C content of 35.72 mol% (Zhang et al., 2021). A complete, gapless, circular genome assembly was generated for isolate SP3-1 yielding a 4,035,760 bp genome (Fig. S1, Table S1) with a total G+C content of 35.1 mol% (Heng et al., 2019). Predictions from annotated genome assemblies suggest that strain NS-1^T^ encodes 3,671 proteins and SP3-1 encodes 3,729 proteins. Results from an extensive genome comparison between isolates SP3-1 and NS-1^T^ reveal nucleotide sequence identities between homologous genes at: 20–30% for 200 genes; 40–50% for 122 genes; 60–70% for 76 genes; 80–90% for 2,477 genes; and 100% for 470 genes (see Table 1 and Table S2). No plasmids were detected and the origin of duplication in isolate SP3-1 was determined based on GC skew analysis. Despite the determination that isolate SP3-1 and strain NS-1^T^ both fall under the taxon *I. fonsfrigidae*, significant differences exist beyond their genomic profiles.

### Cell morphology, physiology, and biochemistry reveal differences between *I. fonsfrigidae* NS-1^**T**^ and isolate SP3-1 suggesting that they are distinct strains of the same species

**Figure 2:**
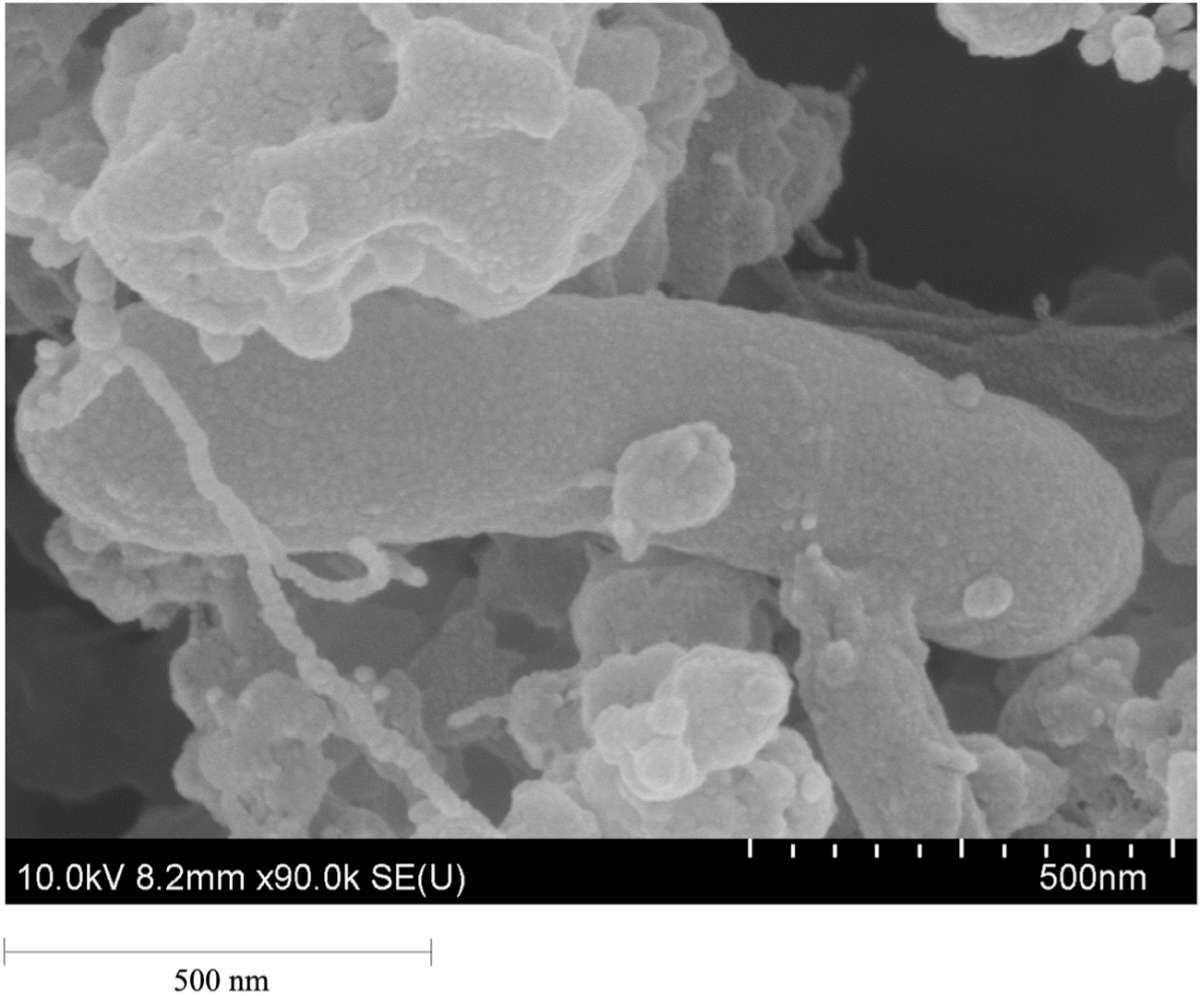
The SEM of *I. fonsfrigidae* strain SP3-1 grown on a basal medium containing 0.5% (w/v) PASC as the sole carbon source with 20% (w/v) NaCl, scale bar = 500 nm.

On solid media, isolate SP3-1 forms small, white colonies with a well-bounded smooth surface. SEM of SP3-1 reveals rod-shaped cells approximately 0.4 µm dia. × 1.3 µm in length (Fig. 2). In contrast electron microscopy of strain NS-1^T^ reveals longer rod-shape cells approximately 0.2–0.3 µm dia. × 6.0–10.0 µm in length. Strain NS-1^T^ also exhibits multiple, long (5–10 µm) flagella extending from one end (*i.e.,* unipolar) of the major axis of the cell (Zhang et al., 2021). Both *I. fonsfrigidae* NS-1^T^ and SP3-1 are Gram-negative and do not form non-endospores. When cultured in BM with 1% (w/v) cellobiose as a carbon source but with different conditions of pH (5.0–10.0) and temperature (25–70 °C), isolate SP3-1 exhibits a wider range of viable growth compared to strain NS-1^T^ (see Table 2). Although optimal growth of isolate SP3-1 occurs at pH 8.0, 37 °C, it readily grows at pH 10.0, confirming that it is a mesophilic alkaliphile. Beyond growth optima, the viable temperature range for isolate SP3-1 is 25–55 °C and the viable pH range is 5.5–10.0 (Table 2). Comparatively, *I. fonsfrigidae* NS-1^T^ grows between 20–45 °C at pH values between 6.5–8.0 with optima at 37 °C and pH 7.0 (Zhang et al., 2021). Moreover, SP3-1 grows on all of the same carbon sources as strain NS-1^T^, except D-rhamnose and alginate, and lignin. Suitable carbon sources include: L-arabinose, D-fructose, D-galactose, D-glucose, D-mannose, D-raffinose, D-xylose, cellobiose, lactose, maltose, sucrose, xylan, and starch (Table 2). We note that strain SP3-1 also readily grows on PASC.

**Table 1:** All genes indicated high similarity and coverage between strain SP3-1 and NS-1^**T**^. The percentage identity of genes>70% indicated high similarity and high coverage.

**Strain**		**SP3-1**	**NS-1** ^**T**^
**High similarity and high coverage genes**		**2933**	**2899**
Identity of genes (%)	90–100	2814	2792
	80–90	80	75
	70–80	39	32
**The number of different genes**		**796**	**772**
**Total genes**		**3729**	**3671**

**Table 2:** Differentiation of the *I. fonsfrigidae* SP3-1 from related members in the family *Halanaerobiaceae* Strains: 1, *Iocasia fonsfrigidae* SP3-1 (this study); 2, *Iocasia fonsfrigidae* NS-1^T^; 3, *Halocella cellulolytica* DSM7362^T^; 4, *Halothermothrix orenii* H168^T^.

**Characteristic**	**1**	**2**	**3**	**4**
Isolation source	Salt evaporation pond	Sediment, cold seep, South China Sea	Hypersaline lagoons of Lake Sivash	Sediment of a Tunisian salted lake
Morphology	Rods	Long rods	Rods	Rods
Cell size (µm)	0. 4 × 1.3	0.2–0.3 × 6.0 −10.0	0.4 −0.6 × 3.8 − 12.0	0.4 −0.6 × 10.0 − 20.0
Flagella	None	Flagella	Flagella	Flagella
NaCl concentration range (%)	5 −30	1.25–15.0	5 −20	4 −20
Optimum NaCl (%)	20	2.5–7.5	15	5 −10
Temperature growth range (° C)	25 −55 (opt. 37)	20–45 (opt. 37)	20 −50 (opt. 39)	45 −68 (opt. 60)
pH growth range	5.5 −10.0 (opt. 8.0)	6.5–8.0 (opt. 7.0)	5.5 −8.5 (opt. 7.0)	5.5 −8.2 (opt. 6.5 −7.0)
Carbon sources (0.5%)				
L-Arabinose	+	+	–	+
D-Fructose	+	+	+	+
D-Galactose	+	+	–	+
D-Glucose	+	+	+	+
D-Mannose	+	+	NA	–
D-Raffinose	+	NA	+	+
D-Rhamnose	–	+	NA	NA
D-Xylose	+	+	–	+
Cellobiose Lactose Maltose	+ + +	NA NA +	+ + NA	+ + -
Sucrose	+	+	+	–
PASC	+	NA	NA	NA
Xylan	+	+	–	NA
Starch	+	+	+	+
Alginate	–	+	NA	NA
Lignin	–	+	NA	NA
Antibiotic sensitivity	Ampicillin (100 µg/mL), ceftriaxone (60 µg/mL), ciprofloxacin (4 µg/mL), clindamycin (10 µg/mL), rifampicin (50 µg/mL), and vancomycin (30 µg/mL),	Ampicillin (100 µg/mL), erythromycin (20 µg/mL) and rifampicin (50 µg/mL)	^a∗^ Ampicillin (100 µg/mL), amikacin (8 µg/mL), amoxycillin (5 µg/mL), ceftriaxone (60 µg/mL), ciprofloxacin (4 µg/mL), clindamycin (10 µg/mL), kanamycin (100 µg/mL), rifampicin (50 µg/mL), trimethoprim (20 µg/mL), and vancomycin (30 µg/mL)	NA
Antibiotic resistance	Amikacin (8 µg/mL), amoxicillin (5 µg/mL), colistin (8 µg/mL), kanamycin (100 µg/mL), and trimethoprim (20 µg/mL)	Kanamycin (100 µg/mL), gentamicin (20 µg/mL), chloramphenicol (20 µg/mL), streptomycin (30 µg/mL), and vancomycin (30 µg/mL)	^a∗^ Colistin (8 µg/mL)	NA
DNA G+C content (mol%)	35.1	35.72	29.0	39.6
Fermentation products*	Acetate, butyrate, CO_2_, H_2_, ethanol, butanol	Acetate, butyrate, ethanol, lactate, propionate H_2_, CO_2_	Acetate, ethanol, lactate, CO_2_, H_2_,	Acetate, ethanol, CO_2_, H_2_
Reference	This study	Zhang et al. (2021)	Simankova et al. (1993)	Cayol et al. (1994)

**Notes.**

NA, indicates not available; “+,” growth; “ ”, no growth; “ *”, used glucose as carbon source, “a*” purchase strain DSM 7362^T^ to culture for check the antibiotic sensitivity and resistance; Opt. is optimum.

The main metabolic products of isolate SP3-1 are acetic acid, butyric acid, carbon dioxide, hydrogen, ethanol, and butanol (Table 2). *I. fonsfrigidae* NS-1^T^ produces lactate when grown on glucose (Zhang et al., 2021); however, isolate SP3-1 does not produce lactate during growth on glucose. One of the most distinguishing features of isolate SP3-1 is that is readily grows in high salt. Specifically, isolate SP3-1 grows in 5–30% (w/v) NaCl with an optimum at 20% (w/v) NaCl. *I. fonsfrigidae* NS-1^T^ grows in a salt concentration range of 1.25–15.0% (Zhang et al., 2021). The ability of isolate SP3-1 to grow in a medium containing up to 30% (w/v) NaCl distinguishes it as more halophilic than other related strains, including *I. fonsfrigidae* NS-1^T^.

In addition to morphological and physiological differences between *I. fonsfrigidae* NS-1^T^ and isolate SP3-1 in terms of: size and shape; substrate utilization; and, temperature, pH, and salinity ranges - there are notable differences in antibiotic resistance between the two isolates. SP3-1 is sensitive to ampicillin (100 µg/mL), ceftriaxone (60 µg/mL), ciprofloxacin (4 µg/mL), clindamycin (10 µg/mL), rifampicin (50 µg/mL), and vancomycin (30 µg/mL); however, this isolate is resistant to amikacin (8 µg/mL), amoxicillin (5 µg/mL), colistin (8 µg/mL), kanamycin (100 µg/mL), and trimethoprim (20 µg/mL). This differs from *I. fonsfrigidae* NS-1^T^, which in addition to ampicillin (100 µg/mL) and rifampicin (50 µg/mL) sensitivity, is also sensitive to erythromycin (20 µg/mL). Like isolate SP3-1, *I. fonsfrigidae* NS-1^T^ is resistant to kanamycin (100 µg/mL). Unlike isolate SP3-1, *I. fonsfrigidae* NS-1^T^ is also resistant to gentamicin (20 µg/mL), chloramphenicol (20 µg/mL), streptomycin (30 µg/mL) and vancomycin (30 µg/mL) (Table 2).

Despite genetic similarity indicating that *I. fonsfrigidae* NS-1^T^ and isolate SP3-1 are the same species, the numerous macromorphological, physiological, and biochemical differences suggest that isolate SP3-1 is not merely a geographic variant but indeed a distinct strain of *I. fonsfrigidae*.

### Chemotaxonomic analysis reveals major differences in polar lipid composition between *I. fonsfrigidae* NS-1^**T**^ and isolate SP3-1

To further study differences in strain SP3-1 *versus* NS-1^T^, chemical profiles were investigated. In general, fatty acid profiles are suitable for differentiating between closely related genera. Indeed, it is suggested that quantitative cellular fatty acid composition differentiate species (Tindall et al., 2010). The cell wall peptidoglycan of strain SP3-1 contains meso-diaminopimelic acid (*meso-* DAP) as the diagnostic diamino acid. The cellular fatty acid profile of strain SP3-1 is shown in Table S3. The cellular fatty acid composition of strain SP3-1 reveals: aiC_15:0_ (21.5%), nC_14:0_ (13.8%), iC_14:0_ (12.6%), iC_15:0_ (11.7%), and nC_16__:0_ (11.4%). This differs from the fatty acid composition of many related type strains in the family Halanaerobiaceae. For example, aiC15:0 is absent in *Halothermothrix orenii* H168^T^ (Cayol et al., 1994) and *Haloanaerobium praevalens* GSL^T^ (Zeikus et al., 1983). However, aiC15:0 is present in *Halocella cellulosilytica (* DSM 7362^T^) but only at 7.6% (Simankova et al., 1993). Interestingly, strain NS-1^T^ features an aiC15:0 composition of 23.7% (Zhang et al., 2021), which more closely resembles that of strain SP3-1. Significant variability in predominant cellular fatty acid content among members of the Halanaerobiaceae has been previously reported (Oren, 2015). In the case of polar lipid profiles, strain SP3-1 contains diphosphatidylglycerol (DPG), phosphatidylethanolamine (PE), phosphatidylglycerol (PG), phosphatidyl-*N*-methyl ethanolamine (PME), phosphatidylcholine (PC), an unidentified amino lipid (*i.e.,* AL1), an unidentified polar lipid (*i.e.,* L1), and unidentified glycolipids (GL2, GL3, and GL4) (Fig. S2). Unlike strain SP3-1, the composition of polar lipids was found in strain NS-1^T^ consists only of diphosphatidylglycerol (DPG), phosphatidylglycerol (PG), an unidentified phosphoglycolipid (*i.e.,* PGL), and two unidentified glycolipids (GL) (Zhang et al., 2021).

These cellular fatty acids and polar lipid profiles support the suggestion that strain SP3-1 is the same species as NS-1^T^ while highlighting major biochemical differences between these two different strains. Significantly different polar lipid profiles between members of related (or the same) taxa often indicated adaptation to different niches and thus different functionality (Dlugosch et al., 2022).

### Functional genomics analysis of *I. fonsfrigidae* strain SP3-1 indicates a complex carbohydrate metabolism

To elucidate differences in metabolic potential between *I. fonsfrigidae* NS-1^T^ and strain SP3-1 fully annotated genomes were analyzed to detect clusters of orthologous genes that drive distinct metabolic functions. From the ∼4.0 Mbp genome, 4,044 genes are predicted with 3,875 protein-coding sequences (CDS), 12 rRNA sequences, and 59 tRNA sequences (Table S1). In comparison, for the ∼3.9 Mbp genome of *I. fonsfrigidae* NS-1^T^ 3,774 genes were predicted with 3,671 protein-coding sequences (CDS), 12 rRNA, and 58 tRNA sequences (Zhang et al., 2021). Notably, the NCBI Prokaryotic Genome Annotation Pipeline (version 4.6) was also employed and predicted 3,885 total genes for strain SP3-1 with 3,729 CDS (see Table S1). These differences are due to the distinct algorithms used in the in-house *versus* the NCBI pipeline. For this study, we used values from the in-house pipeline (*i.e.,* 3,875 CDS) for further analyses.

For strain SP3-1 ∼95% (i.e, 3,666 of 3,875) protein-coding sequences were identified as members of COG functional categories (Tables S1 and S4). Conversely, ∼78% (*i.e.,* 2,889 out of 3,671) of the protein-coding sequences of *I. fonsfrigidae* NS-1^T^ fall into COG functional categories. Major COG functional categories identified include: amino acid transport and metabolism (strain SP3-1 and strain NS-1^T^; E:238 and E:174, respectively); translation, ribosomal structure, and biogenesis (strain SP3-1 and strain NS-1^T^; J:144 and J:160); replication, recombination, and repair (strain SP3-1 and NS-1^T^; L:195 and L:176, respectively); transcription (strain SP3-1 and strain NS-1^T^; K:268 and K:284); cell wall/ membrane/ envelope biogenesis (strain SP3-1 and strain NS-1^T^; M:201 and M:189, respectively); and, of interest for this study, carbohydrate transport and metabolism (strain SP3-1 and NS-1^T^; G:439 and G: 365, respectively). In addition to differences in carbohydrate metabolism, a remarkable difference in COG calls with unknown function is present (strain SP3-1 and NS-1^T^; S:1,019 and S:538, respectively). These predicted COGs may represent additional, and perhaps novel, proteins related to carbohydrate metabolism since this category dominates the COG profile (Olsen, Choffnes & Mack, 2012). Annotation of the strain SP3-1 genome reveals 17 CHA enzymes (compared to 9 CHA enzymes for strain NS-1^T^), which justifies further investigation of the carbohydrate degradation potential of strain SP3-1 (Table 3). Indeed, many of the unknown functional genes may be involved in polysaccharide deconstruction processes and the transport of sugar products (Olsen, Choffnes & Mack, 2012; Kaznadzey, Shelyakin & Gelfand, 2017).

**Table 3:** The comparison of genes encoding for cellulolytic, hemicellulolytic, and amylolytic enzymes in the genome of *I. fonsfrigidae* strain SP3-1 and *I. fonsfrigidae* NS-1^**T**^.

**Enzymes**	**EC number**	**Strains**	
** **	** **	**SP3-1**	**NS-1** ^**T**^
**Cellulolytic enzyme**			
*β*-Glucosidase	3.2.1.21	4	2
Endoglucanase	3.2.1.4	2	1
	**Total**	**6**	**3**
**Hemicellulolytic enzyme**			
Endo- *β*-1,4-galactanase	3.2.1.89	1	0
Xylan 1,4- *β*-xylosidase	3.2.1.37	1	1
*β*-Galactosidase	3.2.1.23	1	1
Xylan- *α*-1,2-glucuronosidase	3.2.1.131	1	0
*β*-Xylosidase	3.2.1.37	1	0
*α*-Xylosidase	3.2.1.177	1	1
*α*-L-Arabinofuranosidase	3.2.1.55	1	0
*β*-L-Arabinofuranosidase	3.2.1.185	1	0
	**Total**	**8**	**3**
**Amylolytic enzyme**			
*α*-Amylase	3.2.1.1	1	2
Oligo- *α*-1,6-glucosidase	3.2.1.10	1	0
Pullulanase	3.2.1.41	1	1
	**Total**	**3**	**3**

### *I. fonsfrigidae* strain SP3-1 encodes for and produces CHA enzymes with a notable range of cellulolytic, hemicellulolytic, and amylolytic activities

Both the range of carbon sources promoting viable growth of strain SP3-1 as well as the detection of known (and expected) carbohydrate metabolism-related COGs identified in the strain SP3-1 genome justify further examination of the CHA enzymes potential of strain SP3-1. To gauge the extent to which strain SP3-1 serves as a suitable prospect for unique carbohydrate deconstruction enzyme discovery, more detailed analyses of genes (and proteins) involved in carbohydrate metabolism was conducted. Because strain SP3-1 grows rapidly on PASC (as well as xylan and starch), a quantitative comparison was performed between strain SP3-1 and an available relative, *H. cellulosilytica* (DSM 7362^T^). *H. cellulosilytica* DSM 7362^T^ grows slowly on PASC and starch. It does not grow on xylan. Residual PASC (dry weight) values after 3 days of growth for SP3-1 and *H. cellulosilytica* DSM 7362^T^ were 46.53% and 75.73%, respectively (Fig. S3).

**Figure 3:**
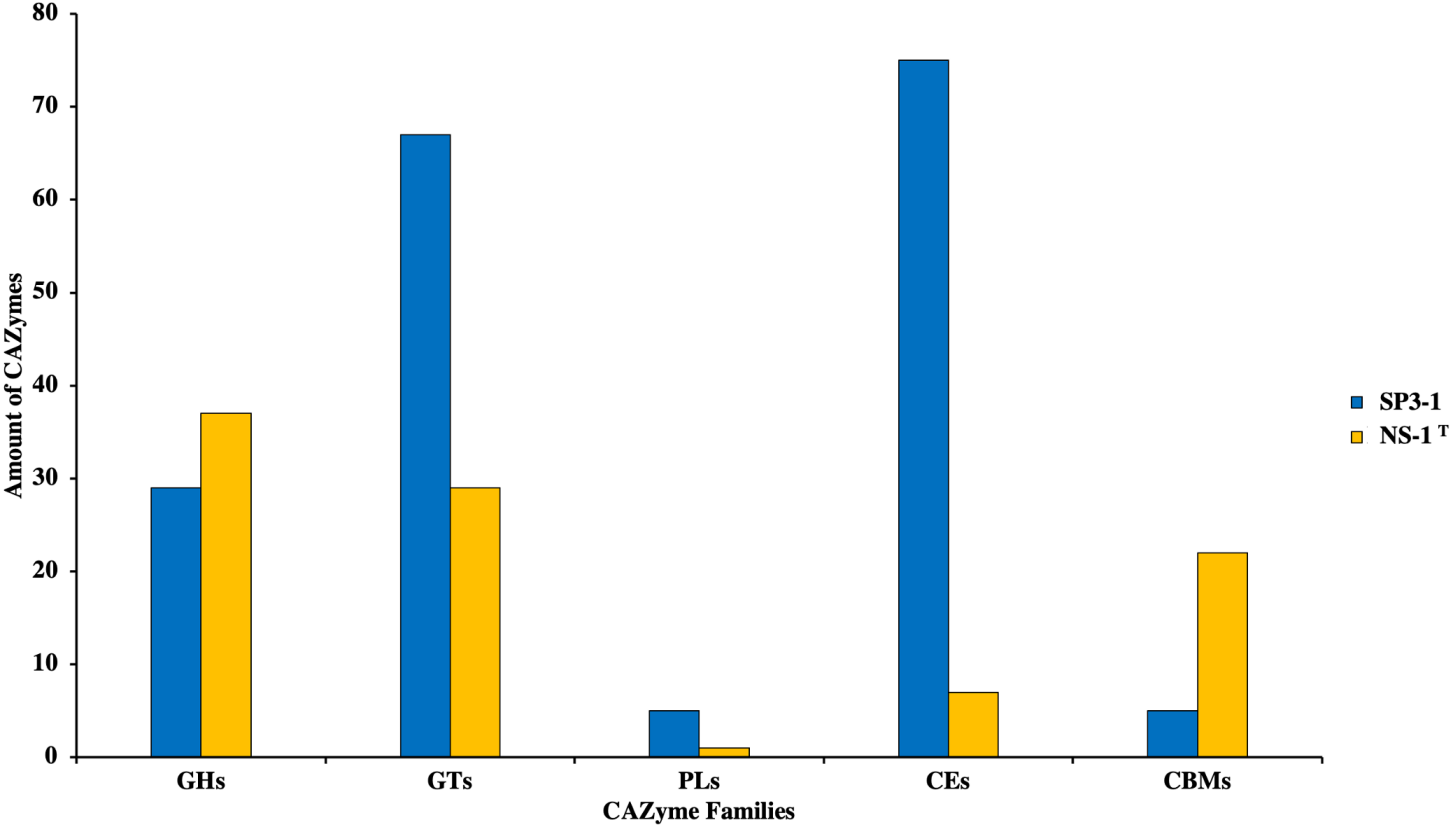
Comparative analysis of CAZymes in *I. fonsfrigidae* strain SP3-1 and *I. fonsfrigidae* NS-1^T^. Number of CAZymes in both strains and their distributions among different families. GHs, glycoside hydrolases; GTs, glycosyl transferases; PLs.

Growth on PASC is important since many industrial-use feedstocks are acid pretreated either prior to enzymatic hydrolysis steps of biomass deconstruction. Carbohydrate Active Enzymes (CAZymes) are involved in the breakdown, biosynthesis, and/or modification of glycoconjugates, oligosaccharides, and polysaccharides (Yang et al., 2019). On swollen cellulosic substrates, endoglucanase (and *β*-glucosidases) are key to effective cellulose substrate deconstruction. Although *I. fonsfrigidae* NS-1^T^ was not available for side-by-side quantitative comparison of PASC utilization, it is noted that strain SP3-1 has at least double the number of endoglucanases and *β*-glucosidases (see Table 3). Indeed, results from advanced COG analysis reveal 181 genes in SP3-1 that are likely involved in carbohydrate breakdown. This includes genes encoding: GHs (glycoside hydrolases, 29), GTs (glycosyl transferases, 67), PLs (polysaccharide lyase, 5), CEs (carbohydrate esterases, 75), and CBMs (carbohydrate-binding modules, 5) (see Fig. 3). CAZymes in *I. fonsfrigidae* NS-1^T^ are about half as many. Specifically, *I. fonsfrigidae* NS-1^T^ has only 96 genes encoding for CAZymes including: GHs, GTs, PLs, CEs, and CBMs (Fig. 3).

Several CHA enzymes are found in the genome of *I. fonsfrigidae* strain SP3-1 (Table S5). Cellulolytic enzymes were found in the *I. fonsfrigidae* strain SP3-1 loci: AZO94579.1 and AZO94980.1 (endoglucanases); and, AZO93138.1, AZO94802.1, AZO96211.1, and AZO96438.1 (*β*-glucosidases). Hemicellulolytic enzymes were detected in the genome of strain SP3-1 at loci: AZO93139.1 (*β*-xylosidase), AZO94803.1 (*β*-galactosidase), AZO95768.1 (*α*-xylosidase), AZO96253.1 (xylan- *α*-1,2-glucuronosidase), AZO96255.1 (xylan 1,4- *β*-xylosidase), AZO96369.1 (endo- *β*-1,4-galactanase), AZO96448.1 (*α*-L-arabinofuranosidase), and AZO96465.1 (*β*-L-arabinofuranosidase) (see Table S5). Amylolytic enzymes are also encoded in the *I. fonsfrigidae* strain SP3-1 genome, including genes at loci: AZO93170.1 (*α*-amylase), AZO93840.1 (oligo- *α*-1,6-glucosidase), and AZO96695.1 (pullulanase), (Table S5). These are endo-acting, exo-acting, and debranching amylases, respectively. When compared to *I. fonsfrigidae* NS-1^T^, strain SP3-1 has a higher number of CHA genes suggesting higher potential for starch-based biomass deconstruction.

To determine the fecundity of *I. fonsfrigidae* strain SP3-1 was cultivated in the BM containing PASC (0.5% w/v) as a carbon source at pH 8.0 and 37 °C. Culture supernatant was harvested after 3 days (at late exponential growth phase) and concentrated. The crude supernatant concentrates exhibit cellulase activity of 5.86 U/g protein on PASC. This CHA gene expression appears to be inducible. In addition, xylanase (4.43 U/g protein) and amylase (4.71 U/g protein) activities were also detected (Table 4). These are likely constitutive enzymes involved in the breakdown of xylan and starch, respectively. Thus, supernatant concentrate derived from strain SP3-1 cultures contains CHA enzymes which readily degrade PASC, xylan, and starch, which are present in the starch-based biomass.

**Table 4:** Enzymatic activities of the crude enzyme from *I. fonsfrigidae* strain SP3-1 grown on the BM containing 0.5% (w/v) PASC as a carbon source.

**Enzyme**	**Specific activity (U/g protein)**
Cellulase	5.86 ± 0.03
Xylanase	4.43 ± 0.04
Amylase	4.71 ± 0.02

## Discussion

Starch-based biomass is a by-product of agro-industrial operations worldwide with millions of tons produced annually (Ceballos, 2017). This biomass consists predominantly of polysaccharides such as cellulose, hemicellulose, and starch—all of which can be used as low-price raw materials to produce secondary products. These substrates can be degraded by enzymes. Enzymatic deconstruction of these polysaccharides can be an “environment-friendly” approach to process feedstock by reducing the use of hazardous chemicals (*e.g.*, strong acids and bases) for biomass conversion (Ceballos et al., 2015; Ceballos, 2017). Specifically, cellulose, hemicellulose, and starch are hydrolyzed by CHA enzymes to yield simple sugars (*i.e.,* monosaccharides and oligosaccharides). Thus, this type of biomass may be used as a renewable resource to produce high-value-added products (Cheawchanlertfa et al., 2021) as long as effective, low-cost enzymes and enzyme technologies are available (Ceballos et al., 2014). Bioprospecting for enzymes capable of efficiently degrading starch-based biomass is an ongoing scientific endeavor and extremophiles are one focus for novel enzyme discovery. Recently, a bacterium strain NS-1^T^ was isolated from deep-sea cold seeps in the South China Sea. The strain NS-1^T^ was reported to have a high similarity to *H. cellulosilytica* DSM 7362^T^. Ultimately, this halophilic isolate NS-1^T^ showed phylogenetic, genomic, and physiological traits unique enough to establish a novel genus within the family Halanaerobiaceae. Thus, isolate NS-1^T^ became the archetype of *I. fonsfrigidae*. *I. fonsfrigidae* NS-1^T^ exhibited the ability to metabolize a diverse array of carbohydrates (Zhang et al., 2021). In this study, the archetype *I. fonsfrigidae* NS-1^T^ (Zhang et al., 2021) and related species were contrasted with the more halophilic *I. fonsfrigidae* strain SP3-1, which was initially designated *Halocella* sp. SP3-1 (Heng et al., 2019).

### Morphological differences between *I. fonsfrigidae* NS-1^**T**^ and strain SP3-1 underscore divergent adaptations in locomotive strategies

Despite the fact that our data support a recent report identifying strain SP3-1 as the same species as *I. fonsfrigidae* NS-1^T^ based on accepted thresholds for genetic similarity (Zhang et al., 2021), we note remarkable differences between strain NS-1^T^ and strain SP3-1 in terms of morphology, physiology, and biochemistry. In terms of morphological differences, the most notable feature of strain SP3-1 (when compared to strain NS-1^T^) is the absence of developed and functional flagella. Although the genome of strain SP3-1 contains many genes related to formation of the basal body of flagella (*i.e.,* *fliE, fliF, fliG, fliH, fliI, fliJ, fliK, fliL, fliM, fliN, fliO, flip, fliQ, fliR, and flhB*) and hook proteins (*i.e.,* *flgA, flgB, flgC, flgD, flgF, flgG, flgH,* and *flgI*), it is missing *flhA* and *flgJ*, which are key genes for basal body and hook formation, respectively (see Table 5 and Table S6). Importantly, the absence or interruption of the *flhA* gene in Gram-negative bacteria leads to nonmotile cells, which lack flagella and are incapable of exporting flagella-related proteins (Bange et al., 2010). The absence of the *flgJ* gene prevents proper assembly of the hook-filament junction in flagella. The *flgJ* gene product is also critical for stabilizing protein-protein interactions between basal structures (*e.g.*, L-ring formation) in flagella (Cohen & Hughes, 2014).

**Table 5:** Genes present only one strain, indicating the differences in phenotypes between strain SP3-1 and NS-1^**T**^.

**SP3-1**	**Length**	**Function**	**NS-1** ^**T**^	**Length**	**Function**	**% Identity**
**Genes coding for the flagellum**						
–	–	–	QTL99899.1	679	Flagellar biosynthesis protein FlhA	–
–	–	–	QTL96989.1	327	Peptidoglycan hydrolase FlgJ	–
**Genes coding for lactate utilization**						
AZO93337.1	233	GntR family transcriptional regulator: HTH-type transcriptional regulator genes (*lutR)*	QTL99594.1	231	PFAM Bacterial regulatory proteins, gntR family	98.68
AZO93433.1	229		QTL99676.1	229	Transcriptional regulator (GntR)	96.07
AZO93498.1	244		–	–	–	–
AZO93635.1	244		–	–	–	–
AZO93658.1	239		–	–	–	–
AZO93770.1	228		QTL96707.1	228	Transcriptional regulator (GntR)	97.36
AZO94208.1	231		QTL97135.1	231	SMART regulatory protein GntR HTH	99.56
AZO94455.1	244		QTL97393.1	220	Transcriptional regulator, GntR family	99.09
AZO94576.1	125		–	–	–	–
AZO94675.1	379		QTL97604.1	358	PFAM regulatory protein GntR HTH	98.60
AZO95757.1	234		–	–	–	–
AZO95845.1	226		–	–	–	–
AZO95907.1	237		–	–	–	–
AZO96195.1	232		QTL98974.1	232	SMART regulatory protein GntR HTH	100
AZO96359.1	243		–	–	–	–
**Genes coding for the Raffinose degradation**						
AZO94804.1	742	Alpha-galactosidase	–	–	–	–
**Genes coding for D-Rhamnose degradation**						
AZO93797.1	332	Rhamnose ABC transporter substrate-binding protein (*RhaS*)	QTL96735.1	332	Rhamnose ABC transporter substrate-binding protein (*RhaS*)	98.44
AZO93795.1	335	ABC tansporter permease (*RhaP*)	QTL96733.1	335	ABC transporter permease (*RhaP*)	98.81
AZO93796.1	328	ABC transporter permease (*RhaQ*)	QTL96734.1	328	ABC transporter permease (*RhaQ*)	98.48
–	–	–	QTL96732.1	505	ATP-binding cassette domain-containing protein rhamnose transport system (*RhaT*)	–
**Genes coding for salt stress/tolerance**						
AZO94203.1	259	Molybdate ABC transporter substrate-binding gene (*modA*)	–	–	–	–
AZO94204.1	268		–	–	–	–
AZO94733.1	155	Glycine/sarcosine/betaine reductase complex selenoprotein A	–	–	–	–
AZO94734.1	441		–	–	–	–
AZO94735.1	339		–	–	–	–
**Genes coding for alkaliphilic**						
AZO93678.1	430	Na^+^/H^+^ antiporter genes	QTL96621.1	430	Na^+^/H^+^ antiporter genes	99.30
AZO93681.1	435		QTL96624.1	345		99.77
AZO94987.1	401		QTL97942.1	401		99.5
AZO95163.1	427		QTL98098.1	427		98.12
AZO96039.1	238		–	–		–
AZO94275.1	517	Na^+^/H^+^ antiporter (NhaC)	–	–	–	–
AZO96817.1	476		–	–	–	–
–	–	–	QTL98140.1	463	Na^+^/H^+^ antiporter (NhaC)	–

**Notes.**

“– ” gene absent in the other strains.

### Physiological enhancements demonstrate the ability of strain SP3-1 to survive and thrive in a broader range of environments than strain NS-1^**T**^ and other related species

Beyond the ability of strain SP3-1 to robustly grow at higher salt concentration and higher pH (when compared to NS-1^T^ and other members of the family Halanaerobiaceae), strain SP3-1 grows on PASC, which makes it a viable candidate for bioprospecting carbohydrate-degrading enzymes. The ability of strain SP3-1 to grow in alkaline environments while utilizing an acid-treated substrate as a carbon source underscores the strain’s tolerance to pH as well as its halophilic nature. This physiological profile is supported by a set of salt stress and high pH tolerance genes identified within the strain SP3-1 genome. For example, the strain SP3-1 genome includes a molybdate ABC transporter substrate-binding gene (*i.e.,* *modA*; gene loci: AZO94203.1, AZO94204.1) (Table 5). The marine bacterium *Staphylococcus* sp. strain P-TSB-70, which readily grows in saline media with up to 20% NaCl (Das et al., 2020), is also endowed with a similar molybdate ABC transporter. Strain SP3-1 also contains three genes encoding a glycine/sarcosine/betaine reductase complex (gene loci: AZO94733.1, AZO94734.1, and AZO94735.1) that are absent in strain NS-1^T^ (Table 5). The glycine/sarcosine/betaine reductase complex includes selenoprotein A. Selenoprotein A is involved in betaine utilization as reported by Manzoor et al. (2015), which demonstrates the growth of selenoprotein A-expressing *Syntrophaceticus schinkii* strain Sp3 growth on betaine. This is notable since accumulation and utilization of betaine from culture media is essential in high salinity conditions as reported by Nyyssölä (2001) in the study of the extreme halophile *Actinopolyspora halophila*.

Na^+^/H^+^ antiporters are also reported to play an essential role in allowing halophilic bacteria to thrive in high salinity environments (Das et al., 2020; Su et al., 2021). Strains SP3-1 and NS-1^T^ both harbor Na^+^/H^+^ antiporter genes; however, strain SP3-1 possesses a larger suite of such genes (gene loci: AZO93678.1, AZO93681.1, AZO94987.1, AZO95163.1, AZO96039.1, AZO94275.1, and AZO96817.1) than NS-1^T^ (see Table 5).

In addition to facilitating salt tolerance, Na^+^/H^+^ antiporters (as well as ABC transporters) are known to be expressed in alkaliphilic halophiles. This has been demonstrated in several species including *B. halotolerans* KKD1 (Cheng et al., 2016), *Bacillus firmus* OF4 (Ito et al., 1997), and *Bacillus* sp. G1 (Liew et al., 2007). The fact that strain SP3-1 is endowed with a more diverse repertoire of genes related to halophilic and alkaliphilic growth suggests a link between genetics and phenotype that permit strain SP3-1 to tolerate higher salt concentrations and higher pH when compared to strain NS-1^T^.

### Genomic analyses highlight genetic substrates that underlie differences carbohydrate metabolism between *I. fonsfrigidae* strains SP3-1 and NS-1^**T**^

Results from carbon utilization studies provided an initial indication that carbohydrate metabolism, biochemistry, and perhaps genetics, are different between *I. fonsfrigidae* strain SP3-1 and NS-1^T^. For example, strain NS-1^T^ grown on glucose yields detectable lactate in the culture supernatant. However, strain SP3-1 does not indicating a difference in sugar metabolism between the strains. Lactate dehydrogenase (LDH) is a key enzyme in the last step of glycolysis that plays a key role in pyruvate-to-lactate reactions (Hamadneh et al., 2021). Although both strains SP3-1 and NS-1^T^ contain LDH genes (gene loci at AZO94114.1 and QTL97032.1, respectively), strain SP3-1 has 15 genes encoding GntR family transcriptional regulators, including an HTH-type transcriptional regulator gene *lutR*. In contrast, strain NS-1^T^ only contains seven GntR genes (see Table 5). The more extensive repertoire of GntR genes may underlie the ability of strain SP3-1 to metabolize lactate. For example, it is known that operons associated with lactate metabolism are controlled by the GntR family (Augustiniene & Malys, 2022). Specifically, HTH-type transcriptional regulators (*lutR* genes) have been shown to regulate genes involved in lactate utilization (Wang et al., 2019). Thus, strain NS-1^T^ with fewer GntR genes may exhibit reduced efficiency in metabolizing lactate, which SP3-1 readily metabolizes this by-product of glucose utilization.

It is notable that SP3-1 can also uptake raffinose as a sole carbon source. This is not surprising since the SP3-1 genome encodes an *α*-galactosidase gene (locus: AZO94804.1), which is absent in the genome of strain NS-1^T^ (see Table 5). It is known that *α*-galactosidase degrades raffinose. This was demonstrated in both *Pseudobalsamia microspore* (Yang et al., 2015) and *Saccharomyces cerevisiae* (Álvarez Cao et al., 2019).

Conversely, strain NS-1^T^ encodes an ATP-binding cassette domain-containing protein rhamnose transport system gene (*i.e.,* *rhaT),* which is lacking in SP3-1. This may underlie the inability of strain SP3-1 to use rhamnose as a carbon source (*see* Richardson, Hynes & Oresnik, 2004).

### Biochemical (and metabolic) properties highlight the CHA enzymes potential of *I. fonsfrigidae* strain SP3-1

Differences in carbohydrate metabolism extend well beyond simple absence/presence of genes for utilization of simple sugars as carbon sources. *I. fonsfrigidae* strain SP3-1 also stands out for its suite of genes encoding CHA enzymes. Not only does the strain SP3-1 genome encode more CHA enzymes than NS-1^T^, but the range of exo-acting, endo-acting, and side chain-acting enzymes are more expansive. The complement of CHA enzymes in strain SP3-1 suggests that its carbohydrate metabolism is more advanced than that of strain NS-1^T^.

Since these classes of enzymes work in concert (and often synergistically) to degrade polysaccharides (Linares-Pasten, Andersson & Karlsson, 2014), the extensive repertoire of CHA enzymes found in the strain SP3-1 genome justifies exploration of this halophilic alkaliphile as a potential source for novel enzyme discovery. Our data on strain SP3-1 show a suite of endoglucanases, which promote cleavage at internal sites within cellulose molecular structures, as well as *β*-glucosidases, which act on short-chain oligosaccharides and cellobiose to produce glucose (Baramee et al., 2017). Genomic data for strain SP3-1 also show a gene encoding for CBM. CBM is reported to assist in the binding of enzymes to insoluble substrates to promote the efficient degradation of cellulosic substrates (Limsakul et al., 2021).

Strain SP3-1 also contains genes encoding for hemicellulolytic enzymes. This suite of enzymes includes: xylan 1,4- *β*-xylosidase, *β*-xylosidase, *α*-xylosidase, xylan- *α*-1,2-glucuronosidase, *α*-L-arabinofuranosidase, *β*-L-arabinofuranosidase, endo- *β*-1,4-galactanase, and *β*-galactosidase (Table S5). Such enzymes are key in the conversion of hemicellulose fractions to simpler sugars (*i.e.,* monomeric carbohydrates). Hemicellulolytic enzymes are useful in biofuels production (Ceballos, 2017) and the synthesis of prebiotics (Phakeenuya et al., 2020).

In addition to cellulolytic and hemicellulolytic enzymes, strain SP3-1 harbors a suite of amylolytic genes that include endo-acting, exo-acting and debranching amylases. Amylolytic enzymes catalyze the cleavage of *α*-D-1,4- and *α*-D-1,6-glycosidic linkages of starch and related oligosaccharides producing short-chain oligosaccharides and glucose (Sidar et al., 2020). Oligosaccharides from starch are used as prebiotics to promote the growth of healthy gut microflora (Belorkar & Gupta, 2016). Furthermore, amylolytic enzymes can be used in starch liquefaction as well as in paper, food, pharmaceutical, and sugar production operations (Pervez et al., 2014).

Since CHA enzymes are the primary enzymes for the breakdown of polysaccharides in starch-based biomass, strain SP3-1 is an attractive and promising microorganism for the novel discovery of CHA enzymes and conversion of starch-based biomass into value-added products.

## Conclusions

*Halocella* sp. SP3-1 was isolated from a high salt evaporation pond in Samut Sakhon, Thailand as described by Heng et al. (2019). The whole-genome sequence of strain SP3-1 was deposited at NCBI GenBank under the accession number CP032760. It was later found to be a species of a new taxon, *Iocasia fonsfrigidae*, which includes a characterized type strain: NS-1^T^ (Zhang et al., 2021). Strain SP3-1, which was isolated from a salt evaporation pond, readily grows in higher salt content of 30% NaCl and higher pH than the type strain NS-1^T^. The halophilic and alkaliphilic nature of strain SP3-1 prompted a studied focused on its potential to metabolize simple and complex carbohydrates as well as the genetic/genomic substrates that underlie its phenotypic nature.

In this study, we have demonstrated that despite the same species determination for strain SP3-1 (Heng et al., 2019) and type strain NS-1^T^ (Zhang et al., 2021), *I. fonsfrigidae* strain SP3-1 is more halophilic and alkaliphilic that strain NS-1^T^ and that there are genetic differences that can account for phenotypic (*e.g.*, morphological and physiological) differences between these two strains. Our analyses demonstrate that strain SP3-1 expresses and secretes a suite of CHA-enzymes which is distinct from that of strain NS-1^T^. Given the adaptation of strain SP3-1, to higher salinity and higher pH environments, this strain serves as a suitable candidate for novel enzyme discovery. Although both strains of *Iocasia fonsfrigidae* are likely limited in their ability to degrade lignocellulosic substrates due to the absence of ligninolytic enzymes such as: lignin peroxidase, manganese peroxidase, versatile peroxidase, laccase, phenoloxidases, and auxiliary enzymes, which play a key role in the degradation of lignin (Biko, Bloom & Van Zyl, 2020), the prospect of discovering or engineering high salt- and high pH- tolerant enzymes from the strain SP3-1 proteome is promising and the subject of ongoing research.

##  Supplemental Information

10.7717/peerj.14211/supp-1 Supplemental Information 1 Circular genome map of *I. fonsfrigidae* strain SP3-1 Marked characteristics are shown from outside to the center; CDS on the forward strand, CDS on the reverse strand, tRNA (light green), rRNA (red), GC (light green peak), content, and GC skew (light green peak describes the region that has a higher G content).

10.7717/peerj.14211/supp-2 Supplemental Information 2 Two-dimensional thin-layer chromatograms of the polar lipids from *I. fonsfrigidae* strain SP3-1 were detected with the following reagents: phosphomolybdic acid (A), Dittmer and Lester (B), ninhydrin (C), anisaldehyde (D), and Dragendorff’s (E). AL1, unidentified aminolipid; DPG, diphosphatidylglycerol; GL2, GL3, GL4, unidentified glycolipids; L1, unidentified polar lipid; PC, phosphatidylcholine; PE, phosphatidylethanolamine; PG, phosphatidylglycerol; PME, phosphatidyl-*N*-methyl ethanolamine.

10.7717/peerj.14211/supp-3 Supplemental Information 3 Use of PASC by *I. fonsfrigidae* strain SP3-1 compared to the closest related species *H. cellulosilytica* DSM7362^T^

10.7717/peerj.14211/supp-4 Supplemental Information 4 Genome features of *I. fonsfrigidae* strain SP3-1 and *I. fonsfrigidae* NS-1^T^.

10.7717/peerj.14211/supp-5 Supplemental Information 5 The complete comparison of all genes between strain SP3-1 and NS-1^T^ with the percent nucleotide sequence identity

10.7717/peerj.14211/supp-6 Supplemental Information 6 Percentage of cellular fatty acid contents of *I. fonsfrigidae* strain SP3-1 from related members in the family *Halanaerobiaceae* Strains: 1, *Iocasia fonsfrigidae* SP3-1 (this study); 2, *Iocasia fonsfrigidae* NS-1^T^; 3, *Halocella cellulolytica* DSM7362^T^;n, normal or straight-chain; i and ai, branched chains; the first number represents the length of the carbon chain, and the second number refers to the number of double bonds.

10.7717/peerj.14211/supp-7 Supplemental Information 7 The cluster of orthologous group (COG) functional categories of the *I. fonsfrigidae* strain SP3-1 and *I. fonsfrigidae* NS-1^T^ enomes

10.7717/peerj.14211/supp-8 Supplemental Information 8 Genes encoding for cellulolytic, hemicellulolytic, and amylolytic enzymes in the genome of *I. fonsfrigidae* strain SP3-1 The bold locus tag indicates the presence of the CBM domain in its structure. The domains were identified using the conserved database domain (NCBI), dbCAN, and InterProScan.

10.7717/peerj.14211/supp-9 Supplemental Information 9 Genes coding for flagellum of the *I. fonsfrigidae* strain SP3-1 and *I. fonsfrigidae* NS-1^T^ ND, indicates not detected

10.7717/peerj.14211/supp-10 Supplemental Information 10 The degradation of strain SP3-1 compared with DSM7362

10.7717/peerj.14211/supp-11 Supplemental Information 11 Raw data of enzymatic activities of the crude enzyme from *I. fonsfrigidae* strain SP3-1

## Additional Information and Declarations

### DNA Deposition

The following information was supplied regarding the deposition of DNA sequences: The 16S rRNA gene sequence of *Iocasia fonsfrigidae* strain SP3-1 is available at GenBank: MW958225. The whole-genome sequence of *Iocasia fonsfrigidae* strain SP3-1 is also available at GenBank: CP032760.

### Data Availability

The following information was supplied regarding data availability: The raw data are available in the Supplemental Files.

### New Species Registration

The following information was supplied regarding the registration of a newly described species: The strain SP3-1 is deposited in Thailand and South Korea under deposit codes TISTR 2992 (Thailand Institute of Scientific and Technological Research) and KCTC 25333 (Korean Collection for Type Cultures), respectively.
