# Supplementary figures and images for: Genomics and cellulolytic, hemicellulolytic, and amylolytic potential of Iocasia fonsfrigidae strain SP3-1 for polysaccharide degradation
Source: PeerJ. 2022 Oct 19;10:e14211. doi: 10.7717/peerj.14211 (PMC9587714; doi:10.7717/peerj.14211)

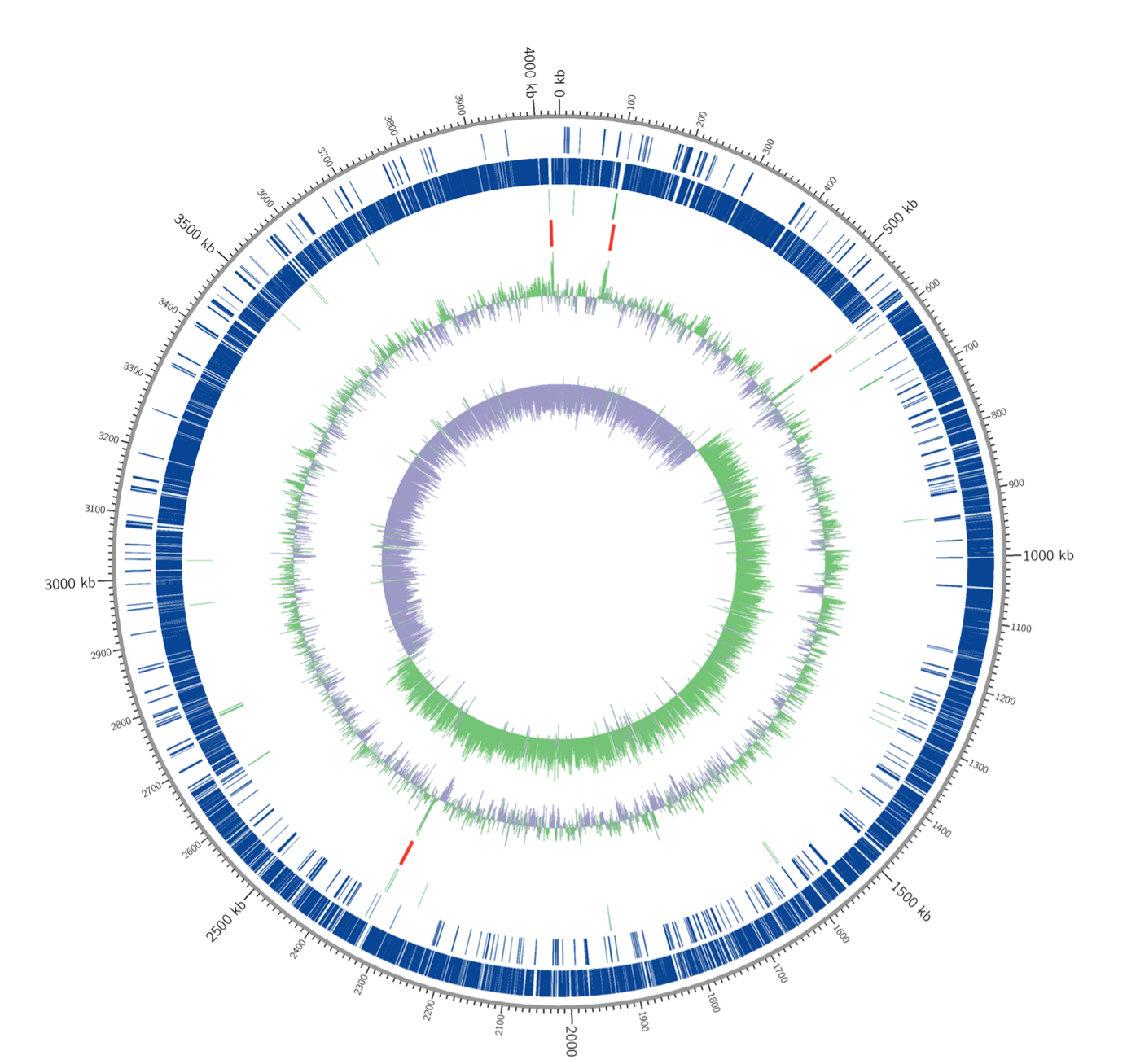

Supplement: Supplemental Information 1 — Marked characteristics are shown from outside to the center; CDS on the forward strand, CDS on the reverse strand, tRNA (light green), rRNA (red), GC (light green peak), content, and GC skew (light green peak describes the region that has a higher G content). [file peerj-10-14211-s001.png]

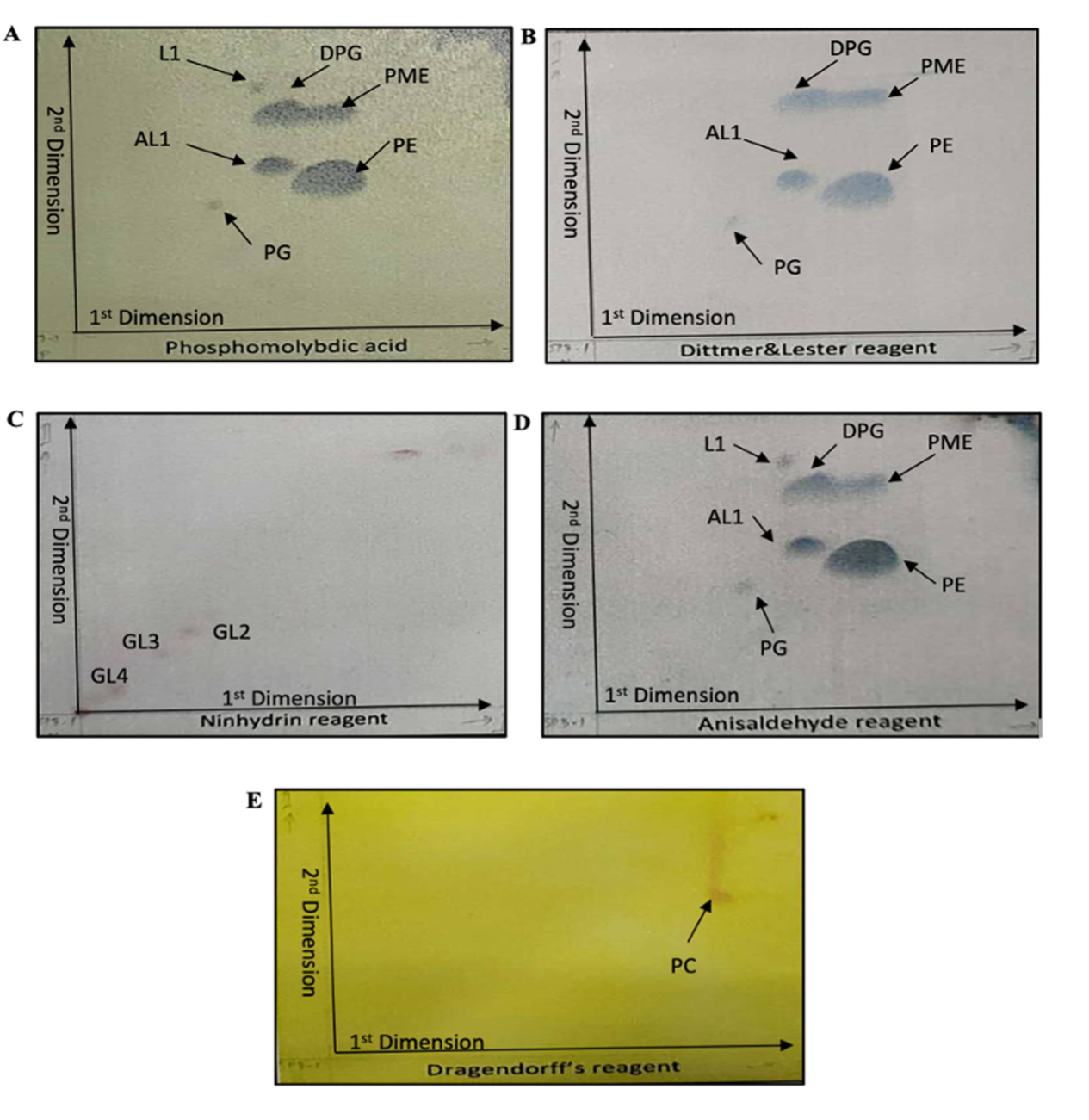

Supplement: Supplemental Information 2 — phosphomolybdic acid (A), Dittmer and Lester (B), ninhydrin (C), anisaldehyde (D), and Dragendorff’s (E). AL1, unidentified aminolipid; DPG, diphosphatidylglycerol; GL2, GL3, GL4, unidentified glycolipids; L1, unidentified polar lipid; PC, phosphatidylcholine; PE, phosphatidylethanolamine; PG, phosphatidylglycerol; PME, phosphatidyl-N-methyl ethanolamine. [file peerj-10-14211-s002.png]

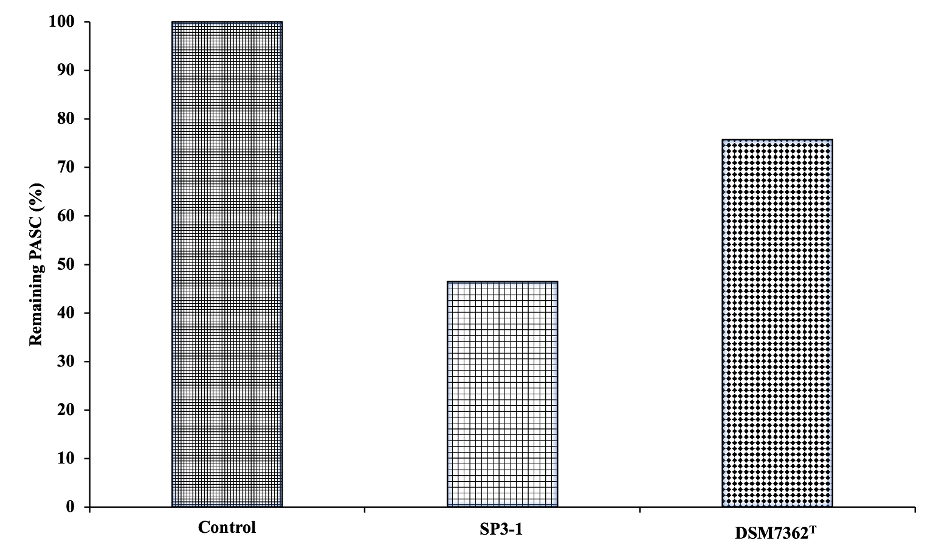

Supplement: Supplemental Information 3 [file peerj-10-14211-s003.png]
