# Supplementary material for: Genomics and cellulolytic, hemicellulolytic, and amylolytic potential of Iocasia fonsfrigidae strain SP3-1 for polysaccharide degradation
Source: PeerJ. 2022 Oct 19;10:e14211. doi: 10.7717/peerj.14211 (PMC9587714; doi:10.7717/peerj.14211)
Supplement: Supplemental Information 4 [file peerj-10-14211-s004.docx]

**Table S1**: **Genome features of *I. fonsfrigidae* strain SP3-1 and *I. fonsfrigidae* NS-1^T^.**

| **Strains** | **Strain SP3-1** | | **NS-1^T^** |
| --- | --- | --- | --- |
| **Features** | **Values** | | |
|  | **In-house pipeline** | **PGAP pipeline** | **PGAP pipeline** |
| Genome size (bp) | 4,035,760 | 4,035,760 | 3,926,493 |
| G + C content (mol%) | 35.1 | 35.1 | 35.72 |
| Total number of genes | 4,044 | 3,885 | 3,774 |
| Protein-coding sequences (bp) | 3,875 | 3,729 | 3,671 |
| rRNA genes | 12 | 12 | 12 |
| tRNA genes | 59 | 59 | 58 |
