# Supplementary material for: Genomics and cellulolytic, hemicellulolytic, and amylolytic potential of Iocasia fonsfrigidae strain SP3-1 for polysaccharide degradation
Source: PeerJ. 2022 Oct 19;10:e14211. doi: 10.7717/peerj.14211 (PMC9587714; doi:10.7717/peerj.14211)
Supplement: Supplemental Information 6 — Strains: 1, Iocasia fonsfrigidae SP3-1 (this study); 2, Iocasia fonsfrigidae NS-1T; 3, Halocella cellulolytica DSM7362T;n, normal or straight-chain; i and ai, branched chains; the first number represents the length of the carbon chain, and the second number refers to the number of double bonds. [file peerj-10-14211-s006.docx]

**Supplemental Table S3:** **Percentage of cellular fatty acid contents of *I. fonsfrigidae* strain SP3-1 from related members in the family *Halanaerobiaceae*** Strains: 1, *Iocasia fonsfrigidae* SP3-1 (this study); 2, *Iocasia fonsfrigidae* NS-1^T^; 3, *Halocella cellulolytica* DSM7362^T^; n, normal or straight-chain; i and ai, branched chains; the first number represents the length of the carbon chain, and the second number refers to the number of double bonds.

| **Chain length** | **1** | **2** | **3** |
| --- | --- | --- | --- |
| aiC12:0 | NA | NA | NA |
| nC12:0 | 0.6 | NA | NA |
| iC13:0 | 2.2 | NA | 0.7 |
| aiC13:0 | 1.1 | NA | 0.4 |
| iC14:1 | NA | NA | 0.5 |
| iC14:0 | 12.6 | NA | 5.2 |
| nC14:1 | NA | NA | 2.1 |
| nC14:0 | 13.8 | 17.9 | 18.0 |
| iC15:1 | NA | NA | 1.0 |
| iC15:0 | 11.7 | 12.0 | 4.8 |
| aiC15:0 | 21.5 | 23.7 | 7.6 |
| nC15:0 | NA | NA | 0.7 |
| nC16:1 | 0.4 | NA | 5.5 |
| iC16:0 | 1.7 | NA | 1.9 |
| nC16:0 | 11.4 | 9.9 | 24.1 |
| iC17:0 | 0.8 | NA | NA |
| aiC17:0 | 0.5 | NA | NA |
| nC17:0 | 0.2 | NA | NA |
| nC18:1 | 0.4 | NA | 0.7 |
| nC18:0 | 0.2 | NA | 2.2 |

Cellular fatty acid contents of *I. fonsfrigidae* NS-1^T^ and *H. cellulosilytica* DSM7362^T^ were cited from Zhang et al., 2021 and Simankova et al.,1993, respectively. NA: indicates not available.
