# Supplementary material for: Genomics and cellulolytic, hemicellulolytic, and amylolytic potential of Iocasia fonsfrigidae strain SP3-1 for polysaccharide degradation
Source: PeerJ. 2022 Oct 19;10:e14211. doi: 10.7717/peerj.14211 (PMC9587714; doi:10.7717/peerj.14211)
Supplement: Supplemental Information 7 [file peerj-10-14211-s007.docx]

**Supplemental Table S4:** **The cluster of orthologous group (COG) functional categories of the *I. fonsfrigidae* strain SP3-1 and *I. fonsfrigidae* NS-1^T^ genomes.**

| **Code** | **Functional annotation** | **Number of genes** | |
| --- | --- | --- | --- |
|  |  | **SP3-1** | **NS-1^T^** |
| **A** | RNA processing and modification | 0 | 0 |
| **B** | Chromatin structure and dynamics | 0 | 0 |
| **C** | Energy production and conversion | 162 | 200 |
| **D** | Cell cycle control, cell division, chromosome partitioning | 31 | 43 |
| **E** | Amino acid transport and metabolism | 238 | 174 |
| **F** | Nucleotide transport and metabolism | 74 | 87 |
| **G** | Carbohydrate transport and metabolism | 439 | 365 |
| **H** | Coenzyme transport and metabolism | 81 | 123 |
| **I** | Lipid transport and metabolism | 49 | 49 |
| **J** | Translation, ribosomal structure, and biogenesis | 144 | 160 |
| **K** | Transcription | 268 | 284 |
| **L** | Replication, recombination, and repair | 195 | 176 |
| **M** | Cell wall/ membrane/ envelope biogenesis | 201 | 189 |
| **N** | Cell motility | 55 | 43 |
| **O** | Post-translational modification, protein turnover, chaperones | 76 | 47 |
| **P** | Inorganic ion transport and metabolism | 238 | 190 |
| **Q** | Secondary metabolites biosynthesis, transport, and catabolism | 22 | 30 |
| **R** | General function prediction only | 144 | 0 |
| **S** | Function unknown | 1,019 | 538 |
| **T** | Signal transduction mechanisms | 150 | 92 |
| **U** | Intracellular trafficking, secretion, and vesicular transport | 24 | 46 |
| **V** | Defense mechanisms | 55 | 53 |
| **W** | Extracellular structures | 0 | 0 |
| **Y** | Nuclear structure | 0 | 0 |
| **Z** | Cytoskeleton | 1 | 0 |
| **Total** | **-** | **3,666** | **2,889** |
