# Supplementary material for: Genomics and cellulolytic, hemicellulolytic, and amylolytic potential of Iocasia fonsfrigidae strain SP3-1 for polysaccharide degradation
Source: PeerJ. 2022 Oct 19;10:e14211. doi: 10.7717/peerj.14211 (PMC9587714; doi:10.7717/peerj.14211)
Supplement: Supplemental Information 8 — The bold locus tag indicates the presence of the CBM domain in its structure. The domains were identified using the conserved database domain (NCBI), dbCAN, and InterProScan. [file peerj-10-14211-s008.docx]

**Table S5**: **Genes encoding for cellulolytic, hemicellulolytic, and amylolytic enzymes in the genome of *I. fonsfrigidae* strain SP3-1.** The bold locus tag indicates the presence of the CBM domain in its structure. The domains were identified using the conserved database domain (NCBI), dbCAN, and InterProScan.

| **Enzymes** | **EC number** | **Length** | **Locus tag** | **Domain organization** |
| --- | --- | --- | --- | --- |
| **Cellulolytic enzyme** | | | |  |
| β-Glucosidase | 3.2.1.21 | 1,352  2,060  1,430  2,483 | AZO93138.1  AZO94802.1  AZO96211.1  AZO96438.1 |  |
| Endoglucanase | 3.2.1.4 | 821  2,549 | AZO94579.1  AZO94980.1 | **AZO94980.1​ ​and GH9-CBM9_1-CBM9** |
| **Hemicellulolytic enzyme** | | | |  |
| Endo-β-1,4-galactanase | 3.2.1.89 | 1,229 | AZO96369.1 |  |
| Xylan 1,4-β-xylosidase | 3.2.1.37 | 2,192 | AZO96255.1 |  |
| β-Galactosidase | 3.2.1.23 | 2,249 | AZO94803.1 |  |
| Xylan-α-1,2-glucuronosidase | 3.2.1.131 | 2,066 | AZO96253.1 |  |
| β-Xylosidase | 3.2.1.37 | 2,471 | AZO93139.1 |  |
| α-Xylosidase | 3.2.1.177 | 2,357 | AZO95768.1 |  |
| α-L-Arabinofuranosidase | 3.2.1.55 | 1,484 | AZO96448.1 |  |
| β-L-Arabinofuranosidase | 3.2.1.185 | 1,961 | AZO96465.1 |  |
| **Amylolytic enzyme** | | | |  |
| α-Amylase | 3.2.1.1 | 1,511 | AZO93170.1 |  |
| Oligo-α-1,6-glucosidase | 3.2.1.10 | 1,637 | AZO93840.1 |  |
| Pullulanase | 3.2.1.41 | 1,946 | AZO96695.1 |  |
