# Supplementary material for: Genomics and cellulolytic, hemicellulolytic, and amylolytic potential of Iocasia fonsfrigidae strain SP3-1 for polysaccharide degradation
Source: PeerJ. 2022 Oct 19;10:e14211. doi: 10.7717/peerj.14211 (PMC9587714; doi:10.7717/peerj.14211)
Supplement: Supplemental Information 9 — ND, indicates not detected [file peerj-10-14211-s009.docx]

**Supplemental Table S6:** **Genes coding for flagellum of the *I. fonsfrigidae* strain SP3-1 and *I. fonsfrigidae* NS-1^T^.**

| **Flagellar Assembly gene** | **SP3-1** | **NS-1^T^** |
| --- | --- | --- |
| Basal body | *fliE* | *fliE* |
|  | *fliF* | *fliF* |
|  | *fliG* | *fliG* |
|  | *fliH* | *fliH* |
|  | *fliI* | *fliI* |
|  | *fliJ* | *fliJ* |
|  | *fliK* | *fliK* |
|  | *fliL* | *fliL* |
|  | *fliM* | *fliM* |
|  | *fliN* | *fliN* |
|  | *fliO* | *fliO* |
|  | *fliP* | *fliP* |
|  | *fliQ* | *fliQ* |
|  | *fliR* | *fliR* |
|  | ND | *flhA* |
|  | *flhB* | *flhB* |
| Hook | *flgA* | *flgA* |
|  | *flgB* | *flgB* |
|  | *flgC* | *flgC* |
|  | *flgD* | *flgD* |
|  | *flgE* | *flgE* |
|  | *flgF* | *flgF* |
|  | *flgG* | *flgG* |
|  | *flgH* | *flgH* |
|  | *flgI* | *flgI* |
|  | ND | *flgJ* |
| Hook-filament junction | *flgK* | *flgK* |
|  | *flgL* | *flgL* |
| Filament | *fliC* | *fliC* |
| Filament cap, chaperone | *fliD* | *fliD* |
|  | *fliS* | *fliS* |
| Regulator | *flgM* | *flgM* |

ND, indicates not detected
